# Polyherbal extract improves glycometabolic control in alloxan-induced diabetic rats *via* down-regulating the MAPK/JNK pathway, modulating Nrf-2/Keap-1 expression, and stimulating insulin signaling

**DOI:** 10.22038/IJBMS.2023.72553.15780

**Published:** 2024

**Authors:** Bilal Aslam, Asif Hussain, Muhammad Naeem Faisal, Shaneel Kousar, Alishbah Roobi, Muhammad Rehan Sajid, Aneela Gul

**Affiliations:** 1 Institute of Physiology and Pharmacology, University of Agriculture, Faisalabad-38040, Faisalabad, Punjab, Pakistan; 2 Department of Pharmacy, Riphah International University, Faisalabad-38000, Faisalabad, Punjab, Pakistan; 3 Department of Pharmacology, Faculty of Pharmacy, University of Lahore-54590, Lahore, Punjab, Pakistan

**Keywords:** Alpha-amylase, Anti-oxidant, Hyperglycemia, Oxidative stress, Polyherbal extract

## Abstract

**Objective(s):**

This study focused on the evaluation of antioxidant and antidiabetic activities of polyherbal extract (PHE), containing *Cassia absus* (L.), *Gymnema sylvestre* (R. Br.), *Nigella sativa* (L.), and *Piper nigrum* (L.), in alloxan-induced diabetes model.

**Materials and Methods:**

*In vitro*, HPLC characterization, DPPH scavenging assay, and α-amylase inhibition test were conducted. *In vivo*, acute oral toxicity of PHE was assessed. Alloxan-induced diabetic Wistar rats (n=6) were orally treated with PHE (200, 400, and 600 mg/kg/day) and glibenclamide (GLB; 10 mg/kg/day) for six consecutive weeks. Then, biochemical biomarkers, oxidative stress parameters, histopathological examination, and mRNA expression levels (RT-qPCR) were determined.

**Results:**

The presence of polyphenols in PHE was confirmed in correlation to marked DPPH scavenging (IC_50_: 1.60 mg/ml) and α-amylase inhibition (IC_50_: 0.82 mg/ml). PHE demonstrated no toxicity in rats up to a dose of 2000 mg/kg. In diabetic rats, PHE dose-dependently ameliorated the serum levels of glucose, insulin, glycated hemoglobin A1c (HbA1c), leptin, and glucokinase (GCK). Also, PHE substantially alleviated serum inflammatory markers (TNF-α and CRP) and oxidative stress indicators (MDA, SOD, and CAT) in pancreatic tissues. PHE, particularly at 600 mg/kg, attenuated cellular oxidative stress *via* modulating the mRNA expression levels of genes regulating MAPK/JNK (Mapk-8, Traf-4, and Traf-6) and Nrf-2/Keap-1 pathways and promoted insulin signaling through up-regulating insulin signaling cascade (Pdx-1, Ins-1, and Ins-2), as compared to GLB. Furthermore, histopathological findings supported the aforementioned results.

**Conclusion:**

Our study suggests that polyherbal extract has promising antioxidant and antidiabetic activities by modulating the MAPK/JNK, Nrf-2/Keap-1, and insulin signaling pathways.

## Introduction

Diabetes mellitus (DM) is a major public health problem across the world, with type-2 DM patients accounting for 90% of all reported cases (1). DM is a complex, multifactorial, and chronic disorder characterized by elevated blood glucose levels (hyperglycemia). It is manifested by an absolute or relative deficiency of insulin production or secretion by pancreatic β-cells, as well as its inability to produce physiological effects (2). Hyperglycemia is a major contributor to oxidative stress, which further disrupts insulin secretion and stimulates the development of insulin resistance (3). Also, compromised anti-oxidant defense mechanisms leave the pancreas particularly sensitive to ROS, leading to the development of diabetes-induced tissue damage *via* redox imbalance and oxidative stress (4).

Therapeutic approaches are centered on improving the body’s glucose usage and metabolism by boosting pancreatic insulin production and sensitivity through the consumption of nutritious foods, diet management, and the use of insulin injections or conventional hypoglycemic agents. These factors promote pancreatic β-cell survival (5)*.* Although there are a variety of commercially available antidiabetic medicines, the side effects associated with these synthetic agents have led to a quest for natural alternative therapies. It is customary to expect traditional herbal therapies to be effective in the prevention and treatment of DM, with fewer side effects (6). As a result, for the management of hyperglycemia, a substantial number of herbs have been favored since they are regarded as safe and more readily available than synthetic agents (7). 

Several plants have demonstrated promising antidiabetic activities in various *in vitro* and *in vivo* investigations (8, 9); hence, plants high in anti-oxidants can be used to manage DM. Therefore, our study investigated four potential antidiabetic plants, including *Cassia absus *L. (Chaksu seed) (10), *Nigella sativa *L. (Black seed) (11), *Piper nigrum *L. (Black pepper) (12), and *Gymnema sylvestre *R. Br. (Cowplant) (13), for the formulation of herbal remedy that may help diabetic patients better regulate their blood glucose levels. Herbal formulations contain several bioactive components, such as alkaloids, saponins, glycosides, phenols, flavonoids, and terpenoids, which have antidiabetic, anti-oxidant, and hypolipidemic properties. These herbal preparations act *via* stimulating β-cell regeneration, promoting insulin synthesis, and peripheral glucose utilization to produce antihyperglycemic effects (14). Also, polyherbal formulations are preferred by traditional medicine practitioners over single medicinal plant usage in the treatment of complicated disorders like diabetes. Additionally, synergistic effects of bioactive compounds in polyherbal formulations result in improved therapeutic results with fewer adverse effects (15, 16). Polyherbal extract (PHE) used in this study was prepared from methanolic extract of seeds of *C. absus*, *N. sativa*, and *P. nigrum*, and the entire plant of *G. sylvestre*, and different *in vitro* and *in vivo* activities were determined to evaluate its efficacy as an anti-oxidant and anti-diabetic formulation.

## Materials and Methods

***Plant materials***

Seeds of *C. absus*, *N. sativa*, and *P. nigrum* and the whole plant of *G. sylvestre* were procured from the botanical garden of the University of Agriculture Faisalabad, Pakistan. Specimens were authenticated by a taxonomist and submitted to the herbarium collection of the Faculty of Basic Sciences, University of Agriculture Faisalabad, Pakistan (voucher no. 21213 for *Cassia absus*, 21214 for *Gymnema sylvestre*, 21215 for *Nigella sativa*, and 21216 for *Piper nigrum*).

***Preparation of*** ***PHE***

The plant materials were shade-dried for 3–4 weeks and coarsely powdered afterward. Then, 150 g of each powdered plant material was combined (17) and soaked in an aqueous-methanolic (70% v/v) solvent mixture for 48 hr. The obtained extract was filtered and the excessive solvent was removed with the help of a rotary evaporator (Heidolph Rotacool, Germany). The extraction yield percentage of concentrated PHE was 13.91%. 

***HPLC analysis of PHE***

HPLC characterization was done using the HPLC instrument (Shimadzu, Japan), which consisted of a UV-visible detector, a C_18_ column (Shim-Pack CDC-ODS), and EZchrom Elite software. Sample preparation and analysis were done according to a previously described method (18). An accurately weighed 50 mg of PHE was dissolved in 40 ml of 60% methanol, acidified with HCl, and boiled at 90 °C for 2 hr. Sample and mobile phase solvents, i.e., A: 6% acetic acid (pH 2.27) and B: 100% acetonitrile, were degassed using an ultrasonic shaker and filtered through a membrane filter (0.22 µm). Analysis was conducted in gradient mode, maintaining the 1 ml/min flow rate of the mobile phase, 27 °C temperature of column, and detection at 280 nm. For the identification and quantification of phytoconstituents, acquired absorbance spectra with retention times were used.

***DPPH assay of PHE***

A previously described method (19) was followed to test the free radical scavenging activity. To prepare stock solutions, 100 g each of PHE and ascorbic acid (standard) were dissolved in methanol. Whereas the blank solution contained only pure methanol. Various concentrations (0.30–1.5 mg/ml) of mixtures were mixed with 3 ml of DPPH (2,2-diphenyl-1-picrylhydrazyl) of 0.5 mg/ml concentration, and incubated for 30 min. Then, absorbance was checked at 517 nm using a spectrophotometer (Shimadzu , Japan). Percentage values of DPPH inhibition were determined using the given formula, and the obtained results were utilized to calculate IC_50_ values. 

***α*** ***-*** ***Amylase*** ***assay of PHE***

*In vitro,* the antidiabetic potential of PHE was checked by estimating the *α-*amylase inhibition ability (20). Acarbose was employed as a standard reagent. Briefly, PHE and acarbose at different concentrations (0.3–1.5 mg/ml) added to phosphate buffer (0.02 mM, pH 6.8) were mixed with *α-*amylase (0.5 mg/ml). Mixtures were further added to 100 µl of 1% starch solution. A reaction mixture containing all the above-mentioned reagents, except the enzyme, was used as a control. Test and control mixtures were combined with 250 µl of dinitro-salicylic acid reagent, and absorbance was noted at 540 nm. The given formula was applied to find the inhibitory activity, and percentage results were used to drive IC_50_ values. 

***Animals***

Female Wister rats weighing 160–220 g (4 to 5 months of age) were procured from the Animal Breeding Facility, Institute of Physiology and Pharmacology, University of Faisalabad, Pakistan. Animals were acclimatized under optimal laboratory conditions such as a 12-hr light and 12 hr dark cycle, 50–60% relative humidity, and 27±1°C room temperature. Rats were provided a conventional pellet diet and clean drinking water *ad libitum*. The Institutional Biosafety and Bioethical Committee of the University of Agriculture Faisalabad, Pakistan, considered and formally approved all experimental protocols, including animal handling (D. No. 3507/ORIC).

***Acute oral toxicity study of PHE***

Assessment of *in vivo* toxicity of PHE was executed according to OECD-425 guidelines. Rats were divided into normal and test groups (n=6). Normal control was given normal saline (5 ml/kg, PO). PHE was administered with increasing doses, i.e., 300, 500, 1000, and 2000 mg/kg (PO) in test groups. All groups were monitored daily for behavioral changes, toxicity symptoms, and mortality for two weeks.

***Diabetes induction *** Thirty-six rats were chosen and divided into two groups based on body weight. The normal group contained six rats (n=6). The second group of 30 rats intended for diabetes induction was given a single injection of freshly prepared alloxan monohydrate (120 mg/kg, IP) after an overnight fast (21). To prevent hypoglycemia, rats were given a 5% glucose solution *ad libitum* for the following 72 hr. Rats that showed a basal blood glucose level of 250 mg/dl or above were divided for further experimental procedures. 

***Oral glucose tolerance test (OGTT) of PHE***

On the third day, after confirmation and selection of diabetic rats, an OGTT was conducted (22). One group (n=6) without alloxan injection was kept as a normal group. Diabetic rats were weighed and allotted to five groups (n=6). The basal blood glucose levels were measured by the tail-tipping method using a digital glucometer (OnCall*Plus II*). Following that, diabetic rats were given three graded doses of PHE (200, 400, and 600 mg/kg, PO) (23) and 10 mg/kg (PO) of glibenclamide (GLB) (24) as standard antidiabetic drug. Then, rats were given an oral glucose load (2 g/kg) 30 min after the administration of treatments, and blood glucose levels were assessed at 30, 60, and 120 min, respectively.

***Experimental design***

Rats were allotted to six groups, each containing six rats (n=6), and orally treated for six weeks. The normal group (healthy rats) and diabetic group (untreated diabetic rats) received 5 ml/kg of normal saline. Four groups of diabetic rats were treated daily with PHE at 200, 400, and 600 mg/kg (23) and GLB at 10 mg/kg (24).

***Blood and organ sampling***

After six weeks of treatment, rats fasted overnight, and blood samples were collected in gel clot activator tubes through a heart puncture under the influence of moderate anesthesia (3% sodium pentobarbital, IP). Blood samples were incubated for 30 min before being centrifuged at 3000 rpm for 15 min. The separated sera were kept at -20 °C for biochemical analysis. Pancreatic tissues were collected and stored for investigation of oxidative stress markers, histopathology, and RT-qPCR.

***Assessment of biochemical parameters***

The commercially available calorimetric and ELISA kits were used to measure the serum levels of glucose (cat#GLU1473/3, SBio, Spain), insulin (cat#INS5628, Calbiotect, USA), glycated hemoglobin A1c (HbA1c; cat#992547, QCA, Spain), leptin (cat#ab100773, Abcam, UK), glucokinase (GCK; cat#E-EL-R0426, Elabscience-Biotech, China), tumor necrosis factor-α (TNF-α; cat# E0764Ra, Bioassay Technology, China), and C-reactive protein (CRP; cat#E0053Ra, Bioassay Technology, China). The manufacturer’s protocols were followed. Analyses were performed using a spectrophotometer (MultiSkan GO, SkanIt software 4.1). 

***Estimation of oxidative stress markers***

Small portions of the pancreas were minced and added to an ice-cold phosphate buffer of pH 7.4 to prepare tissue homogenate (10%, w/v). The Mixtures were centrifuged at 3000xg for 15 min, and collected supernatants were divided into aliquots. Malondialdehyde (MDA) levels were quantified by following the thiobarbituric acid reacting substances (TBRAS) method. The absorbance of prepared samples was taken at 532 nm and presented as nmol/mg protein (25). Superoxide dismutase (SOD) activity in the pancreas was assessed by measuring the rate of pyrogallol auto-oxidation inhibition at 420 nm and mentioned as U/mg protein (26). The H_2_O_2 _reduction was monitored using a spectrophotometer at 240 nm for the determination of catalase (CAT) activity and expressed as U/mg protein (27).

***Histopathological examination***

For histopathological analysis, small portions of rat pancreata were cut and fixed in a 10% formalin buffer solution (pH 7.4). Then tissues were dehydrated, embedded in paraffin, and sliced to 5–6 μm of thickness with the help of a microtome. Prepared sections were stained with H&E dyes, and histopathological changes were observed using a light microscope (IRMECO GmbH & Co, Germany). An in-built camera was used to capture images at the magnification power of 40 x 10.

***Gene expression analysis***

The RT-qPCR analysis was performed to observe the relative mRNA expression levels of genes, including Mapk-8, Traf-4, Traf-6, Nrf-2, Keap-1, Pdx-1, Ins-1, and Ins-2, regulating cellular oxidative stress and insulin secretion. Total RNA in pancreatic tissues was extracted by adopting the TRIzol method, and contents were determined using a Nanodrop spectrophotometer. The cDNA synthesis kit (cat#679029, Thermo-Scientific, UK) was used for reverse transcription. A list of primers (oligonucleotides) designed using the Gene bank and Primer blast is provided in Supplementary Table 1. The RT-qPCR was conducted using 2 µl of Maxima SYBR GREEN/ROX PCR Master Mix 2X (cat#896415, Thermo-Scientific, UK), 1 µl of oligo-primers (Macrogen, USA), and 7 µl of nuclease-free water (cat#AM9932, Ambion, USA) on the iQ5 Bio-Rad system. The 2^−∆∆Ct ^method was applied for the determination of relative mRNA expression levels of selected genes, using β-actin as a housekeeping gene.

***Statistical analysis***

The data acquired from this investigation were presented as Mean±Standard deviation (SD). Data were statistically analyzed using one-way and two-way ANOVA and Tukey’s test by GraphPad Prism software (version 6.0, CA, USA). Mean differences were assumed significant at the 5% level of significance, i.e., *P*<0.05. 

## Results

***HPLC characterization of PHE***

Table 1 depicts the polyphenol fingerprints of PHE. HPLC findings confirmed the presence of several phytocompounds, presumably quercetin > gallic acid > cinnamic acid > benzoic acid > vanillic acid > syringic acid > *p*-coumaric acid (Supplementary Figure 1), with retention times ranging from 3.283 to 24.917 min. 

***In vitro anti-oxidant and antidiabetic activities of PHE***

The free radical scavenging ability of PHE was assessed through the DPPH assay. Based on the concentration utilized, the findings clearly showed that extract suppressed free radical formation in a dose-dependent manner when compared to ascorbic acid (standard), as shown in Table 2. PHE inhibited DPPH by 55.29±1.78% at the maximum concentration of 1.5 mg/ml, with an IC_50_ value of 1.60 mg/ml compared to 0.94 mg/ml of ascorbic acid.

The *α*-amylase inhibitory activity of PHE from 0.3 to 1.5 mg/ml concentrations was determined to assess *in vitro* antidiabetic activity. PHE demonstrated significant *α*-amylase inhibition (82.71±1.24%), with an IC_50_ value of 0.82 mg/ml. Acarbose (standard) indicated inhibition of *α*-amylase by 91.78±2.01% at the maximum concentration of 1.5 mg/ml (Table 2).

***In vivo toxicity investigation***

Results of acute toxicity revealed that PHE did not demonstrate any significant alteration in behavioral patterns, symptoms of toxicity, or mortality throughout a 2 week observation period. The study was conducted in rats at dosages ranging from 300 to 2000 mg/kg of PHE and was determined to be safe up to the maximum dose of 2000 mg/kg. As a result, PHE was deemed safe for further pharmacological testing within the specified range.

***Oral glucose tolerance test (OGTT)***

**Figure 1:**
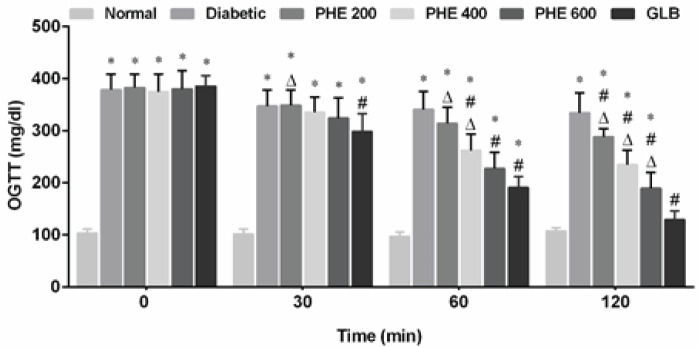
Effect of graded doses of PHE on oral glucose tolerance (OGTT) of alloxan-induced diabetes in rats during the acute study

The impact of PHE on the OGTT of alloxan-induced diabetes in rats is shown in Figure 1. Persistently elevated blood glucose levels were seen in the diabetic group after the oral administration of glucose load (*P*<0.05). The PHE administered at 200, 400, and 600 mg/kg reduced hyperglycemia in diabetic rats in a dose-dependent manner. When compared to the diabetic group, these reductions were significant (*P*<0.05) in PHE (400 and 600 mg/kg) and GLB-treated rats at 60 min and 120 min.

***Effect of PHE on glycometabolic parameters***

After six weeks of diabetes induction by alloxan, a significant (*P*<0.05) increase in glucose and HbA1c and a reduction in insulin were noticed in the sera of the diabetic group as compared to the normal group. Treatment with different doses of PHE appreciably (*P*<0.05) reduced the glucose and HbA1c levels and improved insulin levels in a dose-dependent manner. Moreover, significantly (*P*<0.05) increased leptin and decreased GCK levels were observed in the diabetic group. However, PHE dose-dependently normalized the serum levels of these parameters (*P*<0.05), as shown in Table 3. Furthermore, PHE at 600 mg/kg demonstrated ameliorative effects on these parameters comparable to GLB. 

***Effect*** ***of PHE*** ***on serum inflammatory markers***

**Figure 2:**
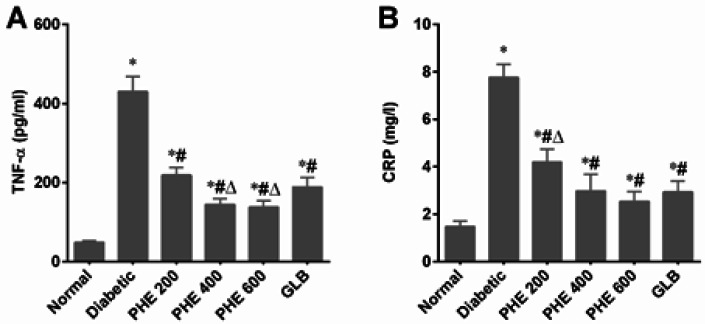
Effect of PHE on inflammatory markers in sera of alloxan-induced diabetic rats

The serum levels of inflammatory markers in diabetic rats were dramatically increased after six weeks of diabetes induction, as presented in Figure 2A, B. A noteworthy (*P*<0.05) increase in TNF-α and CRP levels was noticed in the diabetic group, in contrast to the normal group. Treatment of diabetic rats with PHE at 200, 400, and 600 mg/kg and GLB caused a considerable (*P*<0.05) decrease in TNF-α and CRP levels. Furthermore, it was found that PHE at 400 and 600 mg/kg exerted better attenuating effects as it lowered the TNF-α and CRP levels as compared to GLB-treated diabetic rats.

***Effect of PHE on oxidative stress markers***

**Figure 3:**
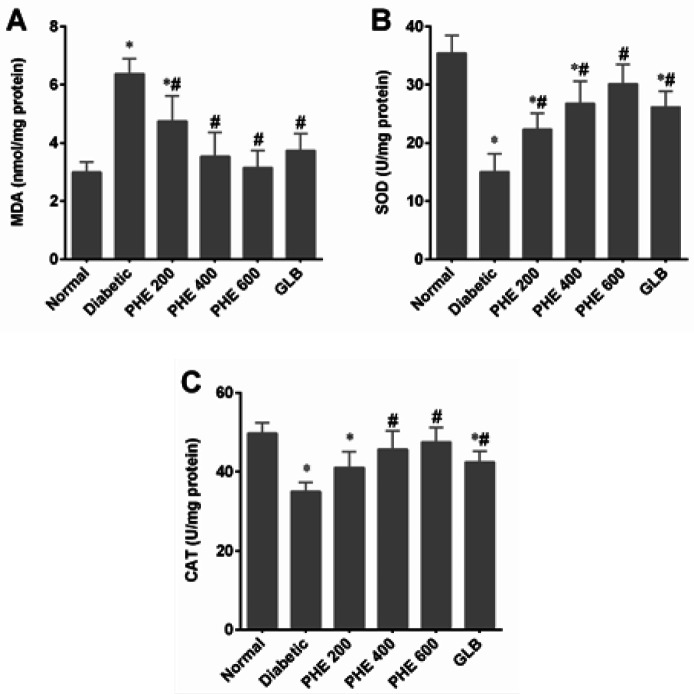
Effect of PHE on oxidative stress parameters in pancreatic tissue of alloxan-induced diabetic rats

Results mentioned in Figure 3 indicate the attenuating effects of PHE on lipid peroxidation (MDA) and anti-oxidant status (activities of SOD and CAT) in pancreatic tissues of diabetic rats. A significant (*P*<0.05) elevation in MDA level and reduction in SOD and CAT activities in the diabetic group was observed after six weeks of diabetes induction. Daily administration of PHE at graded doses significantly (*P<*0.05) lowered MDA levels and enhanced SOD and CAT activities in pancreatic tissues of diabetic rats. As compared to GLB, PHE at 400 and 600 mg/kg exhibited profound effects on lowering MDA levels and raising SOD and CAT activities (Figure 3A-C).

***Histopathological findings***

**Figure 4:**
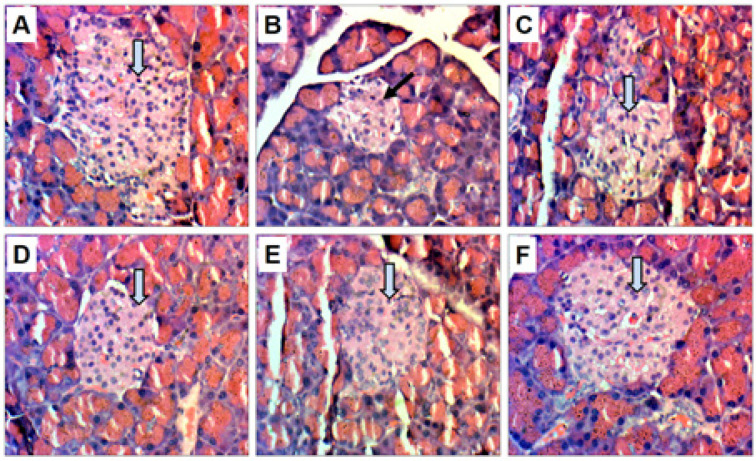
Effect of graded doses of PHE on histopathological changes in the pancreas of alloxan-induced diabetes in rats (magnification: x400)

Histopathological examination of the pancreas of normal rats demonstrated a normal histoarchitecture of islets of Langerhans, normal arrangement of the pancreatic lobule, and connective tissue septa (Figure 4A). However, an excessive disturbance in the cellular arrangement in islets of Langerhans and distortion of pancreatic lobule was noticed in the histology of diabetic group rats in contrast to normal rats (Figure 4B). Treatment of diabetic rats with PHE for six weeks dose-dependently prevented further tissue damage induced by alloxan (Figure 4C-E), particularly at the high dose of 600 mg/kg that was comparable to GLB (Figure 4F).

***Effect of PHE on MAPK downstream JNK signaling***

**Figure 5:**
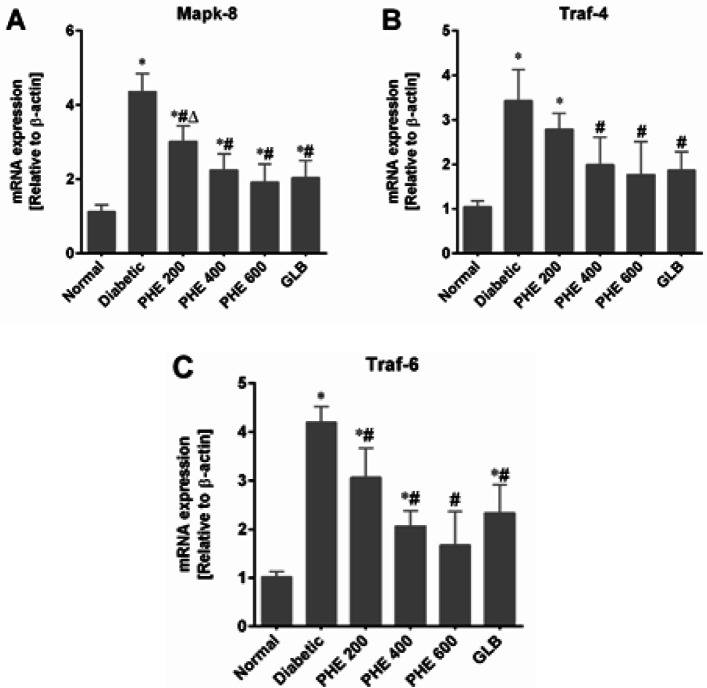
PHE down-regulated the gene expressions of MAPK downstream JNK signaling cascade in diabetic rat

As shown in Figure 5, a marked (*P*<0.05) increase in Mapk-8, Traf-4, and Traf-6 expressions was noticed in the pancreatic tissues of the diabetic group, in contrast to the normal group. The PHE given at graded doses down-regulated the Mapk-8 (*P*<0.05), Traf-4 (*P*<0.05), and Traf-6 (*P*<0.05) expressions in diabetic rats in comparison to the diabetic group. Moreover, PHE at 400 and 600 mg/kg showed a prominent effect on down-regulating Mapk-8, Traf-4, and Traf-6 expressions as compared to GLB (*P*>0.05), as shown in Figure 5(A-C).

***Effect of PHE on Nrf-2 and Keap-1 expressions***

**Figure 6:**
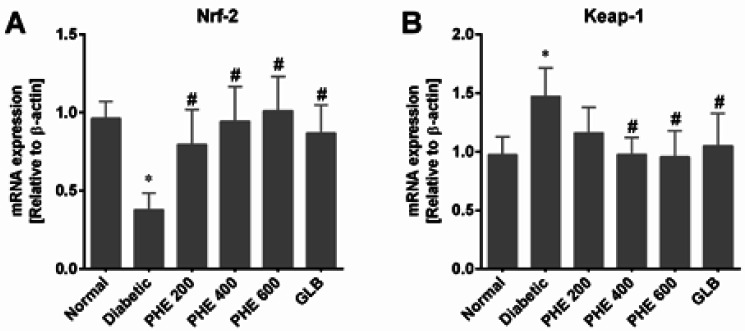
PHE modulated the expressions of Nrf-2 and Keap-1 in diabetic rats

Figure 6 shows the expression levels of Nrf-2 and Keap-1 in the pancreatic tissues of rats after six weeks of diabetes induction. A considerable (*P*<0.05) down-regulation of Nrf-2 and an upsurge of Keap-1 was found in the diabetic group, in contrast to the normal group. Administration of PHE dose-dependently modulated the Nrf-2 (*P*<0.05) and Keap-1 (*P*<0.05) expressions in diabetic rats. In addition, PHE demonstrated better modulatory effects at 400 and 600 mg/kg doses than at 200 mg/kg (Figure 6A, B), and a non-significant (*P*>0.05) difference from GLB was observed.

***Effect of PHE on the insulin signaling pathway***

**Figure 7:**
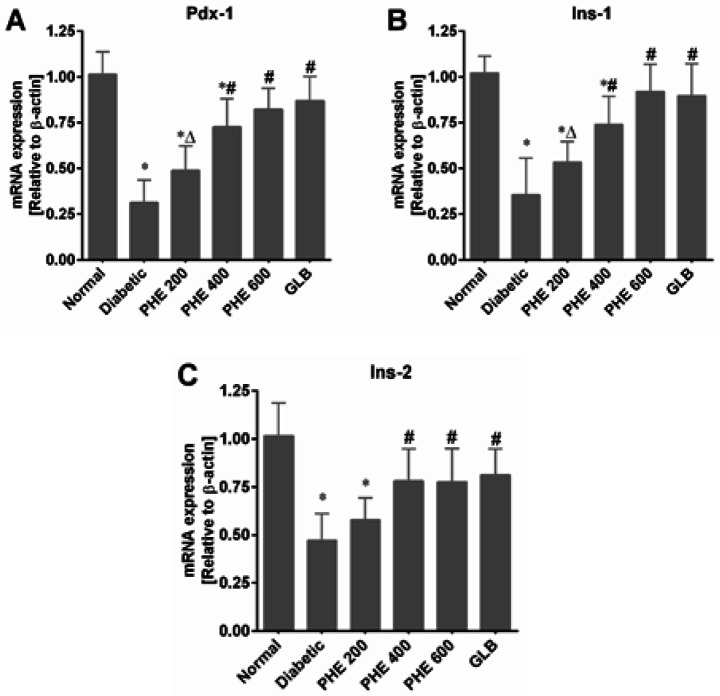
PHE up-regulated the gene expressions of insulin signaling pathways in diabetic rats

The mRNA expression levels of Pdx-1, Ins-1, and Ins-2 involved in the insulin signaling pathway were determined by RT-qPCR. Figure 7 depicts that alloxan caused a significant (*P*<0.05) reduction in expression levels of Pdx-1, Ins-1, and Ins-2 in the diabetic group in comparison to the normal group. The PHE-treated diabetic rats showed a substantial (*P*<0.05) increase in the Pdx-1, Ins-1, and Ins-2 expressions, as compared to the diabetic group. Additionally, a non-significant (*P*>0.05) effect of PHE on expression levels of Pdx-1, Ins-1, and Ins-2 was noticed at 200 mg/kg (Figure 7A-C). Moreover, PHE at 400 and 600 mg/kg exhibited promising effects that were comparable to GLB (*P*>0.05).

**Table 1:** Phytocompounds identified in PHE using HPLC analysis

Retention time (min)	Phytocompounds	Concentration (ppm)
3.283	Quercetin	24.69
4.633	Gallic acid	17.64
13.717	Vanillic acid	11.34
14.267	Benzoic acid	13.56
16.117	Syringic acid	4.92
18.033	*p*-Coumaric acid	4.45
24.917	Cinnamic acid	17.25

HPLC: High-performance liquid chromatography; PHE: Polyherbal extract

**Table 2:** Free radical scavenging and α-amylase inhibitory activities of PHE

Concentration (mg/ml)	DPPH inhibition		α-Amylase inhibition	
	Ascorbic acid (%)	PHE (%)	Acarbose (%)	PHE (%)
0.30	27.81±1.87	16.92±1.65	49.01±1.74	37.12±1.53
0.60	44.03±1.90	21.26±1.12	61.28±2.14	48.83±1.41
0.90	58.27±1.95	29.41±1.24	68.80±1.89	60.22±1.96
1.20	72.38±1.68	43.78±1.85	84.39±2.52	73.29±2.30
1.50	79.49±1.53	55.29±1.78	91.78±2.01	82.71±1.24
IC_50 _value	0.94	1.60	0.46	0.82

Triplicate values are mentioned as mean±SD

PHE: Polyherbal extract

Data are presented as mean±SD, n=6. *P<0.05, normal *vs* other group; #*P*<0.05, diabetic *vs* treated groups; P<0.05, GLB *vs* PHE-treated groups

GLB: Glibenclamide; PHE: Polyherbal extract

**Table 3:** Effect of PHE on serum levels of glycometabolic parameters in alloxan-induced diabetic rats

Groups	Glucose (mg/dl)	Insulin (µIU/ml)	HbA1c (%)	Leptin (ng/ml)	GCK (ng/ml)
Normal	106.49±9.96	16.74±1.82	4.61±0.94	1.18±0.21	5.48±0.85
Diabetic	446.11±28.18*	7.32±1.23*	9.22±1.26*	3.61±0.25*	2.14±0.45*
PHE 200	238.76±10.98*^#^	8.98±2.03*	7.33±1.04*^#^	2.76±0.45*^#^	3.44±0.75*^#^
PHE 400	172.76±15.81*^#^	12.76±2.44*^#^	7.24±1.03*^#^	2.05±0.33*^#^	3.91±0.64*^#^
PHE 600	141.31±12.97*^#^	14.43±1.71^#^	6.57±0.82*^#^	1.82±0.34*^#^	4.73±0.64^#^
GLB	117.81±14.73^#^	14.96±2.49^#^	5.30±0.79^#^	1.42±0.25^#^	5.01±0.50^#^

Data are presented as mean±SD, n=6. *P<0.05, normal *vs* other group; #*P*<0.05, diabetic *vs* treated groups; P<0.05, GLB *vs *PHE-treated groups

HbA1c: Glycated hemoglobin A1c; GCK: Glucokinase; GLB: Glibenclamide; PHE: Polyherbal extract

(A) Tumor necrosis factor-α (TNF-α) and (B) C-reactive protein (CRP). Data are presented as mean±SD, n=6. *P<0.05, normal *vs* other group; #*P*<0.05, diabetic *vs* treated groups; *P*<0.05, GLB *vs* PHE-treated groups

GLB: Glibenclamide; PHE: Polyherbal extract

(A) Malondialdehyde (MDA) level, (B) Superoxide dismutase (SOD) activity, and (C) Catalase (CAT) activity. Data are presented as mean±SD, n=6. *P<0.05, normal *vs* other group; #*P*<0.05, diabetic *vs* treated groups; *P*>0.05, GLB *vs* PHE-treated groups. GLB: Glibenclamide; PHE: Polyherbal extract

(A) Normal group showing intact islet of Langerhans with abundant β-cells. (B) The diabetic group displayed a significant decrease in the number of β-cells, distortion of the islet, and focal necrosis (thin arrow). (C-E) Treatment with PHE at 200, 400, and 600 mg/kg indicated a dose-dependent restoration of the histological structure of the pancreas; an increase in the number of β-cells, and moderate improvement in an endocrine mass of the pancreas (Bold arrow). (F) GLB treatment showing mild histopathological changes in the pancreas. GLB: Glibenclamide; PHE: Polyherbal extract

The mRNA expression levels of (A) Mapk-8, (B) Traf-4, and (C) Traf-6 in pancreatic tissue of alloxan-induced diabetic rats are presented as mean±SD, n=6. *P<0.05, normal *vs* other group; #*P*<0.05, diabetic *vs *treated groups; P<0.05, GLB *vs* PHE-treated groups

GLB: Glibenclamide; PHE: Polyherbal extract

The mRNA expression levels of (A) Nrf-2 and (B) Keap-1 in pancreatic tissue of alloxan-induced diabetic rats are presented as mean±SD, n=6. *P<0.05, normal *vs* other group; #*P*<0.05, diabetic *vs* treated groups; *P*>0.05, GLB* vs* PHE-treated groups

GLB: Glibenclamide; PHE: Polyherbal extract

The mRNA expression levels of (A) Pdx-1, (B) Ins-1, and (C) Ins-2 in pancreatic tissue of alloxan-induced diabetic rats are presented as mean±SD, n=6. *P<0.05, normal *vs* other group; #*P*<0.05, diabetic *vs* treated groups; *P*<0.05, GLB *vs* PHE-treated groups

GLB: Glibenclamide; PHE: Polyherbal extract

## Discussion

A hyperglycemic environment is associated with excessive ROS production in diabetes (3). Alloxan, which was employed in our study to induce diabetes, is known to be converted into dialuric acid, resulting in the formation of ROS and, as a result, the partial necrosis of pancreatic β-cells. This lowers insulin levels, consequently in type 2 DM development, with some remnant pancreatic β-cells possessing insulin-producing potential (28), as seen in a trial using hypoglycemic drugs such as sulphonylureas in alloxan-induced diabetic rats (29). This study aimed to confirm the ethnopharmacological value of PHE by evaluating its phytochemical composition, anti-oxidant activity, possible *in vivo* toxic effects, and antidiabetic efficacy in an alloxan-induced rat model of diabetes.

The phytochemical examination of PHE indicated considerably high concentrations of bioactive phytocompounds in the tested PHE, including quercetin, gallic acid, cinnamic acid, benzoic acid, syringic acid, vanillic acid, and p-coumaric acid (Table 1). Quercetin possesses potent anti-oxidant and anti-inflammatory potential (30). Vanillic acid and syringic acid reduce oxidative damage and inflammation caused by hyperglycemia (31,32). Gallic acid stimulates insulin secretion and possesses anti-inflammatory and anti-oxidant properties (33). Cinnamic acid is reported to improve diabetes control and its complications (34). Hence, all of the phytoconstituents detected in PHE may act synergistically to attenuate hyperglycemia and oxidative stress in diabetic rats.

*In vitro*, PHE showed significant DPPH inhibition, possibly due to the high anti-oxidant capability of detected phytoconstituents to scavenge free radicals (Table 2) as shown in previous studies (9, 10). The α-amylase inhibition test was used to assess the anti-diabetic potential of PHE *in vitro*. The α-amylase is a carbohydrate-digesting enzyme that is essential for the hydrolysis of polysaccharides. PHE at various concentrations significantly decreased the activity of the α-amylase enzyme (Table 2), showing that it could effectively decrease postprandial sugar levels *via* inhibiting the pancreatic α-amylase. The OGTT is a procedure used to assess changes in carbohydrate metabolism following the administration of a glucose load (35). PHE effectively managed glucose levels, subsequently reduced hyperglycemia (Figure 1), and indicated that rats given graded doses of PHE have efficient glucose utilization abilities.

In this study, we employed alloxan to induce diabetes, which preferentially destroys pancreatic β-cells and leads to hyperglycemia (Table 3). Hyperglycemia impaired glucose homeostasis, resulting in substantial (*P*<0.05) elevated glucose and HbA1c levels and lowered insulin levels in diabetic rats. However, a significant decrease in glucose and HbA1c, as well as enhanced insulin levels, anticipate the PHE’s glucose-lowering potential, which might be attributed to the anti-hyperglycemic action of identified phenolics (21). Leptin is a key hormone that regulates energy balance, reduces appetite, and improves glucose consumption and insulin sensitivity under normal physiological conditions. However, in pathological situations such as obesity and hyperglycemia, considerably high levels of leptin have been found, which might be due to leptin resistance (36). Glucokinase (GCK) is an enzyme that controls carbohydrate breakdown or accumulation by shifting cell activity in response to changes in glucose levels (37). In diabetic rats, we found a marked (*P*<0.05) rise in leptin and a drop in GCK levels. PHE significantly improved the diabetes state in a dose-dependent way by decreasing leptin and increasing GCK, most likely *via* reducing ROS generation and inflammatory molecules like IL-6 and TNF-α (38).

Insulin resistance is a primary cause of type-2 DM and contributes to DM-associated co-morbidities (39). The acute phase response is a systemic inflammation that occurs in conjunction with hyperglycemia-linked oxidative stress. This response involves the generation of significant quantities of hepatocyte-derived acute phase proteins (ACPs), like CRP, and could be induced by injecting the cytokines TNF-α and IL-6 into experimental animals (40, 41). Figure 2(A, B) depicts a significant (*P*<0.05) rise in TNF-α and CRP levels in diabetic rats, indicating systemic inflammation. The TNF-α and CRP levels in sera were considerably (*P*<0.05) reduced after treatment with graded dosages of PHE, showing the anti-inflammatory action of PHE (42), particularly at 400 and 600 mg/kg doses.

To examine the mechanism of the PHE’s protective effects on pancreatic β-cells, we measured oxidative stress indicators in pancreatic tissues of diabetic rats. Oxidative stress-induced pancreatic injury is a critical event in the dysfunction of β-cells and diabetes development (43). Pancreatic β-cells are particularly sensitive to ROS (44). Alloxan injection and the resulting hyperglycemia caused an excessive generation of ROS in the pancreas, aggravating the development of DM and its complications. We observed that diabetic rats had significantly (*P*<0.05) higher lipid peroxidation and lower SOD and CAT activities in pancreatic tissues (Figure 3A–C). PHE reduced lipid peroxidation and restored the activity of SOD and CAT in pancreatic tissues in a dose-dependent way, particularly at 400 and 600 mg/kg. This anti-oxidant activity might be attributable to polyphenols, which are plentiful in the examined PHE. Polyphenols are potent anti-oxidants that scavenge reactive oxygen and nitrogen species or serve as peroxyl-radical scavengers (45).

In our study, the histological changes of the pancreas of normal, diabetic, PHE, and GLB-treated diabetic rats were evaluated (Figure 4A–E). Alloxan injection induced a variety of histological alterations, including a decrease in the size of islets of Langerhans and a significant drop in pancreatic β-cell count, as observed in earlier studies (46). Both decreased cell mass and function result in inadequate insulin levels in rats. PHE administered in diabetic rats for six weeks dose-dependently improved islet cell and tissue conditions, as evidenced by a marked increase in pancreatic β-cells, and prevented the histological changes, mainly at the high doses. The activity of different phytocompounds present in PHE may reduce hyperglycemia by one of several mechanisms, including repair of β-cell proliferation, stimulation of insulin production, and augmentation of insulin effects (47, 48).

The role of the JNK (c-Jun N-terminal protein kinase) signaling pathway downstream of MAPK has been thoroughly understood in the pathogenesis of DM. In response to stress stimuli such as environmental factors and inflammation, the JNK proteins are stimulated by a cascade of phosphorylation reactions (49,50). Oxidative stress activates TNF receptor-associated factors (Traf-4 and Traf-6) and the consequent stimulation of JNK signaling in different tissues. ROS and JNK may have a positive feedback effect, in which excessive ROS increases gene expression implicated in the JNK pathway and active JNK encourages further ROS generation (51). MapK-8, also called JNK-1, exacerbates oxidative stress, which further leads to the activation of stress-induced apoptosis. Also, it induces inflammation, pancreatic β-cell malfunction, and the secretion of inadequate or faulty insulin from β-cells (52). Our results showed that Mapk-8, Traf-4, and Traf-6 expression levels were considerably (*P*<0.05) overexpressed in diabetic rats (Figure 5A–C). Oral treatment with PHE resulted in a significant (*P*<0.05) drop in ROS levels, which resulted in lower expressions of Mapk-8, Traf-4, and Traf-6 in diabetic rats, particularly at 400 and 600 mg/kg. Furthermore, our findings showed that PHE could inhibit hyperglycemia-linked JNK pathway activation, leading to a marked decrease in ROS and apoptosis of pancreatic β-cells.

The Nrf-2/Keap-1/ARE signaling pathway has been identified as a key anti-oxidant signaling pathway in DM pathogenesis (53). Studies have revealed that therapies targeting the Nrf-2/Keap-1/ARE pathways provide an attractive way of relieving oxidative stress in DM patients (54). Hence, we investigated the anti-oxidant capacity of PHE on the Nrf-2/Keap-1 pathway in diabetic rats by measuring the mRNA expression levels of the Nrf-2 and Keap-1 genes. Diabetes induction caused a considerable reduction in Nrf-2 expression (*P*<0.05) and an increase in Keap-1 expression (*P*<0.05) in the diabetic group (Figure 6A, B), as reported in a previous study (55). Diabetic rats treated with PHE markedly up-regulated Nrf-2 expression while down-regulating Keap-1 expression levels. Results demonstrated the anti-oxidant activity of PHE in a dose-dependent manner by increasing Nrf-2 nuclear translocation and decreasing Keap-1 mRNA expression in pancreatic tissues of rats with alloxan-induced diabetes.

We examined the mRNA expression levels of Pdx-1, Ins-1, and Ins-2 genes to demonstrate the influence of PHE on insulin production and pancreatic β-cell regeneration. The pancreatic and duodenal homeobox-1 (Pdx-1) gene is a key modulator of glucose-stimulated insulin gene (Ins-1 and Ins-2) transcription (56). Damage to pancreatic β-cell induced by oxidative stress or glucotoxicity lowers Pdx-1 expression, subsequently inhibits insulin production, and promotes diabetes development (43). In this study, diabetes induction by alloxan resulted in considerable (*P*<0.05) suppression of Pdx-1 expression and DNA binding capabilities, which subsequently impaired the expression of insulin genes (Ins-1 and Ins-2). Pdx-1, Ins-1, and Ins-2 expressions were markedly enhanced in diabetic rats treated with PHE at graded dosages, resulting in β-cell regeneration and higher insulin release from β-cells (Figure 7A–C), as seen earlier (57).

GLB improved the glycometabolic control and restored histopathological changes of pancreatic tissue in diabetic rats *via *lowering systemic inflammation (TNF-α and CRP), inhibiting oxidative stress through down-regulating the MAPK/JNK pathway and modulating the expressions of Nrf-2/Keap-1, and activating the insulin signaling pathway. Therefore, it could be concluded that the anti-oxidant and antidiabetic effects of PHE, particularly at 400 and 600 mg/kg doses, possibly involve these mechanisms.

## Conclusion

Our findings reveal that PHE rich in a wide range of phytocompounds has anti-oxidant and hypoglycemic impact on alloxanized diabetic rats *via* lowering hyperglycemia-linked oxidative stress *via* inhibiting the stimulated MAPK downstream JNK pathway and activating the anti-oxidant defense mechanism (Nrf-2/Keap-1 pathway). Furthermore, PHE administration up-regulated the expression of genes involved in the insulin signaling pathway, which collectively enhanced the structural and functional integrity of the pancreas. Thus, our study demonstrates the therapeutic importance of PHE in the glycometabolic control of diabetes, paving the way for the formulation of alternative products that may offer less expensive and safer options for the management of diabetes with fewer side effects.

## Authors’ Contributions

B A, A H, and MN F designed the study; A H, S K, A R, MR S, and A G analyzed the data and prepared the draft manuscript; B A and MN F critical revised the paper; B A and MN F supervised the research; B A, A H, MN F, S K, A R, MR S, and A G approval the final version to be published.

## Conflicts of Interest

None.
